# Bidirectional negative relationship between thyrotropin and kidney function during alcohol intoxication in males

**DOI:** 10.3389/fneph.2024.1322791

**Published:** 2024-08-08

**Authors:** Hayrunnisa Unlu, Asmaa Yehia, Khalid Manji, Noah Manji, Andrés M. Treviño-Alvarez, Tommy Cabeza De Baca, Mark A. Frye, Leslie F. Thomas, Osama A. Abulseoud

**Affiliations:** ^1^ Department of Psychiatry and Psychology, Mayo Clinic Arizona, Phoenix, AZ, United States; ^2^ Department of Neuroscience, Graduate School of Biomedical Sciences, Mayo Clinic College of Medicine, Phoenix, AZ, United States; ^3^ Department of Medical Physiology, Faculty of Medicine, Mansoura University, Mansoura, Egypt; ^4^ Barts and The London School of Medicine and Dentistry, Queen Mary University of London, London, United Kingdom; ^5^ Faculty of Medicine and Dentistry, Queen Mary University of London, Victoria, Malta; ^6^ Obesity & Diabetes Clinical Research Section, Phoenix Epidemiology & Clinical Research Branch, National Institute of Health/National Institute on Diabetes and Digestive and Kidney Diseases, Phoenix, AZ, United States; ^7^ Department of Psychiatry, Universidad Autónoma de Nuevo León, Monterrey, Mexico; ^8^ Department of Psychiatry & Psychology, Mayo Clinic, Rochester, MN, United States; ^9^ Division of Nephrology and Hypertension, Department of Internal Medicine, Mayo Clinic in Arizona, Scottsdale, AZ, United States

**Keywords:** intoxication, thyrotropin, kidney function, GFR - glomerular filtration rate, sex difference

## Abstract

**Introduction:**

Despite a well-established direct toxic effect of alcohol on renal cells, there is a salutary dose-dependent effect of alcohol consumption on common laboratory parameters related to kidney performance. Alcohol also impacts thyroid hormones, while thyroid status modulates kidney function. The modulation of kidney parameters with thyrotropin (TSH) and thyroid status indicates a possible interaction between alcohol, kidney, and thyroid functions. This retrospective study was conducted to test the hypothesis that the positive effect of alcohol use on the estimated glomerular filtration rate (eGFR) is mediated by alcohol’s effect on thyroid hormones.

**Methods:**

We reviewed the electronic medical records of 767 hospitalized adult patients free of thyroid disorders who received medical care in the Mayo Clinic Health System from June 2019 through June 2022 and had blood alcohol concentration (BAC), serum TSH, and serum creatinine measured during the hospitalization. We calculated the eGFR using both the re-expressed Modification of Diet in Renal Disease (MDRD II) study equation and the Chronic Kidney Disease Epidemiology Collaboration (CKD-EPI) Creatinine equation.

**Results:**

We found a significant relationship of BAC with eGFR (CKD-EPI) and TSH in males only. BAC had a positive association with eGFR (b = 0.24, p = 0.0001) and negative with TSH (b=-0.17, p = 0.006). The covariance between the two outcomes (eGFR and TSH) was negative (b = -0.12, p = 0.049). The path analyses using the eGFR MDRD II equation were not significant in males, whereas females had no significant path analyses with either of the eGFR equations.

**Discussion:**

We observed that BAC influences both eGFR and TSH, whereas eGFR and TSH influence each other. After considering important covariates (e.g., age, body mass index, diabetes mellitus, cardiovascular disease, chronic kidney disease, and chronic liver disease) and the negative bidirectional effect of TSH and eGFR, a positive impact of BAC on eGFR was observed in males.

## 1 Introduction

The detrimental health effects of excessive alcohol consumption are well known (1). However, there is an intriguing dose-dependent positive association between alcohol use and kidney function that has been reported in several large-scale studies (2–5). Understanding this beneficial effect of alcohol on kidney function despite its direct toxicity on renal cells (6) through oxidative stress (7), lipid peroxidation (8), and ferroptosis (9), among other mechanisms, is challenging. One way to reconcile this apparent contradiction is to consider that alcohol has both direct deleterious and indirect salutary effects through thyroid hormones.

Alcohol has a direct effect on all levels of the hypothalamic-pituitary-thyroid (HPT) axis (10–16), including thyroid gland volume (17–19) and serum concentrations of the thyroid hormones tetraiodothyronine (T4) (20, 21) and triiodothyronine (T3) (22–24), and thyrotropin (TSH) levels (25–27). Moreover, alcohol alters the activities of the thyroid metabolizing enzyme deiodinase type II, which converts prohormone T4 into active T3 (28, 29). As such, both preclinical and clinical studies have demonstrated changes in different thyroid hormones during chronic alcohol use (21–23), acute intoxication (30), early withdrawal (20, 26, 30), and abstinence (24). The results of these studies are inconsistent, possibly due to confounding factors including: A) timing of blood collections (31, 32), B) presence of liver cirrhosis (27, 33), C) cigarette smoking (34–38), and D) cannabis use (39). However, some findings demonstrate increased peripheral conversion of T4 into T3 and suppression of TSH in the setting of alcohol use (10, 11). Interestingly, findings in a rodent model of chronic alcohol use suggested that increases in intracellular T3 concentration in the amygdala may be specifically related to reward mechanisms and the development of behavioral dependence on ethanol (29).

Thyroid status directly affects kidney function. Hypothyroidism promotes a reduced glomerular filtration rate (GFR), and hyperthyroidism promotes an increased GFR (40–46). The elucidation of mechanisms by which hypothyroidism promotes a reduction in GFR is presently incomplete. Reduced renal sodium and chloride reabsorption (47–49), increased capillary permeability (50), decreased plasma volume (51), decreased cardiac output (51), increased vascular resistance (52, 53), decreased expression of renal vasodilators, decreased renal response to vasodilators, and intrarenal vasoconstriction are adaptations observed with hypothyroidism that may variably contribute to reduced renal blood flow and reduced GFR (54). In several case reports, hypothyroidism was demonstrated to be a cause of kidney dysfunction (55–61). Furthermore, TSH correlates negatively with eGFR (62, 63), and administration of recombinant human thyrotropin to patients (n=24) after total thyroidectomy was associated with a significant reduction in eGFR (64). Similarly, a study showed that thyroid hormone replacement therapy attenuated the rate of renal functional decline in chronic kidney disease patients with subclinical hypothyroidism (65). Taken together, it is plausible to hypothesize that alcohol intoxication causes a transient hyperthyroid state with TSH suppression, which promotes an increase in GFR. To test this hypothesis, we reviewed the charts of patients who were admitted during acute alcohol intoxication with blood alcohol concentration (BAC) above the lower limit of detection (10 mg/dL), had serum TSH and creatinine concentrations during the same hospital admission, and were placed on the Clinical Institute Withdrawal Assessment of Alcohol Scale, Revised (CIWA-Ar) protocol for alcohol withdrawal (66).

## 2 Methods

### 2.1 Patients and data collection

This study was approved by the Institutional Review Board (IRB) of the Mayo Clinic (ID#22-008591). At Mayo Clinic Health Systems (MCHS), patient data from EPIC, the electronic medical record (EMR) system, was initially obtained from an electronic data dump by a senior data analyst. Following this, the lead author (HU) verified the EMR and performed data cleaning. Next, the lead author (HU) manually cross-checked individual data points by reviewing patients’ charts, including providers’ notes, progress notes, admission and discharge notes, and laboratory results. Admission diagnoses were collected from the providers’ notes of the first encounter. Active problem lists and active problems noted by providers were used to report medical and psychiatric comorbidities and substance use profiles. Patients (n=844) included in this study, received medical care in the Mayo Clinic Health System (MCHS) from June 2019 to June 2022. We obtained laboratory results for BAC, TSH and serum Creatinine that were collected concurrently all during the first day of hospital admission. They had measured BAC levels higher than the lower limit of detection method (10 mg/dL), and were placed on the CIWA-Ar protocol (66). Blood ethanol concentration was determined using a headspace gas chromatography-flame ionization detection method (67). The following data were extracted from the EMR: demographics, reason for hospitalization, medical and psychiatric comorbidities, hospital course (including intensive care unit (ICU) admission), laboratory studies including BAC, TSH, creatinine, blood urea nitrogen (BUN), and liver enzymes, and all-cause mortality. To remove the potential confounding effect of comorbid thyroid disorders, we performed the statistical analysis after excluding patients with pre-existing thyroid conditions [n=77 (31 males, 46 females)]. To calculate the estimated GFR (eGFR), we used two equations. The first one is the re-expressed Modification of Diet in Renal Disease (MDRD) II study equation [Re-expressed MDRD II equation = 175 × (sCr)^−1.154^ × (Age)^−0.203^ × (0.742 if female) × (1.210 if African American), where SCr = serum creatinine in mg/dL] (68). We also used the Chronic Kidney Disease Epidemiology Collaboration (CKD-EPI) Creatinine equation [CKD-EPI equation = 141 × min (sCr/κ, 1) ^α^ × max (sCr/κ, 1) ^−1.209^ × 0.993^age^ × 1.018 (if female) × 1.159 (if African American), where κ is 0.7 for females and 0.9 for males, α is −0.329 for females and −0.411for males, min indicates the minimum of sCr/κ or 1, and max indicates the maximum of sCr/κ or 1] (69).

### 2.2 Statistical analysis

We expressed continuous data as median and interquartile range (IQR) and categorical data as percentage. We used the Kolmogorov-Smirnov and Shapiro-Wilk normality tests to test for a normal distribution. To compare continuous variables between males and females, we used the Student’s t test and the Mann-Whitney U test for normally distributed and non-parametric data, respectively. We used the chi-square test to compare categorical variables and the Bonferroni correction for pairwise comparison. Pearson’s correlation analysis was used to test the relationship between BAC and both eGFR and TSH after logarithmic transformation of the positively skewed TSH. We performed mediation analysis to investigate the role of TSH as a mediator in the relationship between BAC and eGFR. Mediation analysis tests potential causal effects in a causal chain in which an independent variable (an exposure), represented here by BAC, affects a dependent variable (an outcome), represented here by eGFR, through an intervening variable called the mediator, represented here by TSH. The mediator is affected by an exposure, affects an outcome, and carries the influence of the exposure on the outcome. Mediation analysis tests the direct effect of the exposure on the outcome and the effect of the exposure on the outcome carried by the mediator (70, 71).

We performed the mediation analysis in four steps, in which several regression analyses were conducted. First, we used a simple linear regression to test for the “C path”, representing the effect of BAC (exposure) on eGFR (outcome). Second, we tested for “A path”, representing the effect of BAC (exposure) on TSH (mediator) using simple linear regression. Third, we performed a simple regression analysis to examine the “B path” which represents the effect of TSH (mediator) on eGFR (outcome). We performed the analysis when all the conditions stated by Baron and Kenny were met. If any nonsignificant relationship was detected in the first three steps, mediation is not possible (71). Fourth, using multiple regression analysis, we tested for the “C′ path”, which represents the effect of BAC (exposure) on eGFR (outcome) when TSH (mediator) is controlled. If “C′ path” is insignificant, this means full mediation, and if significant, this means partial mediation. We further adjusted for age, body-mass index, cardiovascular disorders, hepatic disorders, diabetes, renal disorders, and elevated liver enzymes using the SAS software (SAS 9.4; SAS Institute Inc., Cary, NC, USA). Since the traditional mediation model could be biased (72), assuming that the dependent variable (eGFR) does not affect the mediator (TSH), we further performed path analysis via structural equation modelling (SEM) to test the possible bidirectional relationship between eGFR and TSH. The path analysis evaluated whether BAC predicted both eGFR and TSH values, while accounting for the covariance between both outcomes and controlling for age, body-mass index, cardiovascular disorders, hepatic disorders, diabetes, renal disorders, and liver enzymes. Path analyses via SEM were calculated by the LAVAAN package (73) within R statistical software and stratified by sex. Statistical significance was denoted as p < 0.05 (two-sided). Results were presented as standardized coefficients (β).

## 3 Results

### 3.1 Demographics

Following the exclusion of patients identified as having a comorbid thyroid disorder (n=77), the remaining cohort of 767 patients was categorized into two groups based on sex, including 66.6% (n = 511) males and 33.4% (n = 256) females. No significant sex difference in median age, race, or ethnicity was observed. However, there was a significant sex difference in terms of marital status, with a higher proportion of males being single [54.2% (n=277) vs. 42.6% (n=109), P<0.001], while more females were divorced [15.7% (n=80) vs. 24.6% (n = 63), P<0.001]. Employment status showed a difference, as more females were identified as student/employed compared to males [11.9% (n=61) vs. 21.1% (n=54), P<0.001]. Notably, median BMI was found to be significantly higher among males [median (IQR) 27.3 (7.7) vs. 24.8 (8.3), P<0.001] (**Table 1**).

**Table 1:** Demographics.

		All (n=767)	Males [n=511 (66.6%)]	Females [n=256 (33.4%)]	p value
Age [Median (IQR)]		45 (23)	44 (23)	46.5 (24.7)	0.7
Age groups, No. (%) of patients	<40	301 (39.2%)	200 (39.1%)	101 (39.5%)	0.4
	40-65	398 (51.9%)	265 (51.9%)	133 (52%)	0.8
	>=65	68 (8.9%)	46 (9%)	22 (8.6%)	0.8
BMI (kg/m2) [Median (IQR)]		26.6 (8)	27.3 (7.7)	24.8 (8.3)	<0.001
BMI groups, No. (%) of patients	<18.5 (kg/m2)	21 (2.7%)	14 (2.7%)	7 (2.7%)	0.99
	18.5 to <25.0 (kg/m2)	245 (31.9%)	135 (26.4%)	110 (43%)	<0.001
	25.0 to < 30.0 (kg/m2)	212 (27.6%)	160 (31.3%)	52 (20.3%)	0.001
	30.0 to <40.0 (kg/m2)	165 (21.5%)	113 (22.1%)	52 (20.3%)	0.6
	≥40 (kg/m2)	31 (4%)	23 (4.5%)	8 (3.1%)	0.4
	Unknown	93 (12.1%)	66 (12.9%)	27 (10.5%)	0.3
Race, No. (%) of patients	White	661 (86.2%)	433 (84.7%)	228 (89.1%)	0.1
	African American	51 (6.6%)	37 (7.2%)	14 (5.5%)	0.3
	American Indian/Alaskan Native	13 (1.7%)	9 (1.8%)	4 (1.6%)	0.8
	Other	31 (4%)	26 (5.1%)	5 (2%)	0.04
	Unknown	11 (1.4%)	6 (1.2%)	5 (2%)	0.4
Ethnicity, No. (%) of patients	Non-Hispanic	716 (93.4%)	476 (93.2%)	240 (93.8%)	0.8
	Hispanic	25 (3.3%)	18 (3.5%)	7 (2.7%)	0.6
	Other	6 (0.8%)	5 (1%)	1 (0.4%)	0.4
	Unknown	20 (2.6%)	12 (2.3%)	8 (3.1%)	0.5
Marital Status, No. (%) of patients	Single	386 (50.3%)	277 (54.2%)	109 (42.6%)	<0.001
	Married	181 (23.6%)	120 (23.5%)	61 (23.8%)	0.9
	Divorced	143 (18.6%)	80 (15.7%)	63 (24.6%)	<0.001
	Widowed	28 (3.7%)	11 (2.2%)	17 (6.6%)	0.06
	Separated	15 (2%)	13 (2.5%)	2 (0.8%)	0.1
	Life partner	4 (0.5%)	3 (0.6%)	1 (0.4%)	0.7
	Unknown	10 (1.3%)	7 (1.4%)	3 (1.2%)	0.8
Employment status, No. (%) of patients	Student/Employed	115 (15%)	61 (11.9%)	54 (21.1%)	<0.001
	Self-employed	29 (3.8%)	22 (4.3%)	7 (2.7%)	0.3
	Unemployed	268 (34.9%)	183 (35.8%)	85 (33.2%)	0.5
	Retired	92 (12%)	62 (12.1%)	30 (11.7%)	0.9
	Disabled	53 (6.9%)	40 (7.8%)	13 (5.1%)	0.1
	Other	166 (21.6%)	117 (22.9%)	49 (19.1%)	0.2
	Unknown	44 (5.7%)	26 (5.1%)	18 (7%)	0.3

BMI, Body Mass Index.

### 3.2 Reasons for hospitalization

The most common reason for hospital admission was alcohol intoxication [82.6% (n=635)], followed by suicidal ideation [31.8% (n=244)], altered mental status [18.6% (n=143)], trauma [17.7% (n=136)], alcohol withdrawal [15.6% (n=120)], and suicide attempt [13.6% (n=104)]. There was no significant sex difference in admission diagnosis, except males were more commonly admitted due to trauma [21.9% (n=112) vs. 9.4% (n=24), P<0.001], cardiovascular disorders [8.6% (n=44) vs. 3.1% (n=8), P=0.004], and metabolic derangements [5.5% (n=28) vs. 2% (n=5), P=0.02] (**Table 2**).

**Table 2:** Reason for hospitalization.

		All (n=767)	Males [n=511 (66.6%)]	Females [n=256 (33.4%)]	p value
Alcohol intoxication		635 (82.8%)	421 (82.4%)	214 (83.6%)	0.8
Alcohol withdrawal		120 (15.6%)	83 (16.2%)	37 (14.5%)	0.6
Substance intoxication (other than alcohol)		23 (3%)	18 (3.5%)	5 (2%)	0.3
Suicidal ideations		244 (31.8%)	159 (31.1%)	85 (33.2%)	0.6
Suicide attempt		104 (13.6%)	66 (12.9%)	38 (14.8%)	0.5
Homicidal ideations		11 (1.4%)	8 (1.6%)	3 (1.2%)	0.7
Altered mental status		143 (18.6%)	91 (17.8%)	52 (20.3%)	0.4
Trauma		136 (17.7%)	112 (21.9&)	24 (9.4%)	<0.001
Pain related conditions		91 (11.9%)	66 (12.9%)	25 (9.8%)	0.2
Gastrointestinal disorders		57 (7.4%)	38 (7.4%)	19 (7.4%)	0.99
Cardiovascular disorders		52 (6.8%)	44 (8.6%)	8 (3.1%)	0.004
Neurological disorders	Any	60 (7.8%)	41 (8%)	19 (7.4%)	0.9
	Seizures	18 (2.3%)	9 (1.8%)	9 (3.5%)	0.1
Pulmonary disorders		40 (5.2%)	29 (5.7%)	11 (4.3%)	0.5
Metabolic derangements^a^		33 (4.3%)	28 (5.5%)	5 (2%)	0.02
Other reasons including constitutional symptoms^b^		108 (14.1%)	78 (15.3%)	30 (11.7%)	0.2

a Hypo/hyperglycemia, increased lactate, electrolyte imbalance.

b Dizziness, nausea/vomiting, malaise, fever, chills, fatigue, headache.

### 3.3 Comorbid medical and psychiatric conditions

Out of the patient population, 69.5% (n=533) had medical comorbidities. The most common medical comorbidities were cardiovascular [60.1% (n=461)], gastrointestinal [55.8% (n=428)], respiratory [20.2% (n=155)], and neurologic disorders [19.3% (n=148)]. More females than males had iron deficiency anemia [5.5% (n=14) vs. 1.4% (n=7), P=0.002], and malignancy [7.8% (n=20) vs. 2.9% (n=15), P=0.003]. Conversely, more males than females had comorbid hypertension [32.5% (n=166) vs. 23.4% (n=60), P=0.009].

Within the cohort, 70.1% (n = 538) presented with comorbid psychiatric disorders. The most prevalent psychiatric disorders were depression [50.7% (n=389)], anxiety [37.5% (n=288)], and suicidal ideation [14.6% (n=112)]. Females had significantly more psychiatric comorbidities than males [81.3% (n=208) vs. 64.6% (n=330), P<0.001]. Females were more frequently diagnosed with depression [58.6% (n=150) vs. 46.8% (n=239), P=0.002], anxiety disorder [43.4% (n=111) vs. 34.6% (n=177), P=0.02], post-traumatic stress disorder (PTSD) [11.7% (n=30) vs. 6.7% (n=34), P=0.02], borderline personality disorder [10.9% (n = 28) vs. 4.1% (n = 21), P <0.001], and eating disorders [5.5% (n=14) vs. 0.6% (n=3), P<0.001]. Conversely, males were more frequently diagnosed with schizophrenia [3.1% (n=16) vs. 0.4% (n=1), P = 0.01] and antisocial personality disorder [1.8% (n=9) vs. 0%, P=0.03].

The most common comorbid substance use disorders were moderate or severe alcohol use disorder [74.1% (n=568)], tobacco use disorder [35.2% (n=270)], and cannabis use disorder [8.9% (n=68)]. There was no sex difference in comorbid substance use disorders (**Table 3**).

**Table 3:** Medical and psychiatric comorbidities.

			All (n=767)	Males [n=511 (66.6%)]	Females [n=256 (33.4%)]	p value
Medical	Any medical comorbidity		533 (69.5%)	357 (69.9%)	176 (68.8%)	0.8
	Gastrointestinal disorders	Any	428 (55.8%)	291 (56.9%)	137 (53.5%)	0.4
		Hepatic Disorders	186 (24.3%)	132 (25.8%)	54 (21.1%)	0.1
		Other Gastrointestinal disorders	242 (31.6%)	159 (31.1%)	83 (32.4%)	0.7
	Cardiovascular disorders	Any	461 (60.1%)	335 (65.6%)	126 (49.2%)	<0.001
		Hypertension	226 (29.5%)	166 (32.5%)	60 (23.4%)	0.009
		Cardiomyopathy/Heart Failure	35 (4.6%)	28 (5.5%)	7 (2.7%)	0.1
		Other	200 (26.1%)	141 (27.6%)	59 (23%)	0.2
	Neurological disorders	Any	148 (19.3%)	101 (19.8%)	47 (18.4%)	0.7
		Neuropathy	45 (5.9%)	31 (6.1%)	14 (5.5%)	0.9
		Seizure Disorders	51 (6.6%)	35 (6.8%)	16 (6.3%)	0.9
	Iron Deficiency Anemia		21 (2.7%)	7 (1.4%)	14 (5.5%)	0.002
	Other Anemia		80 (10.4%)	53 (10.4%)	27 (10.5%)	0.99
	Respiratory disorders		155 (20.2%)	103 (20.2%)	52 (20.3%)	0.99
	Dermatologic disorders		59 (7.7%)	34 (6.7%)	25 (9.8%)	0.1
	Diabetes Mellitus		67 (8.7%)	52 (10.2%)	15 (5.9%)	0.06
	Renal/Urinary System disorders		106 (13.8%)	75 (14.7%)	31 (12.1%)	0.4
	Malnutrition		18 (2.3%)	10 (2%)	8 (3.1%)	0.3
	Eye Disorders		40 (5.2%)	28 (5.5%)	12 (4.7%)	0.7
	Malignancy		35 (4.6%)	15 (2.9%)	20 (7.8%)	0.003
	Repeating Falls		68 (8.9%)	47 (9.2%)	21 (8.2%)	0.7
	Orthopedic Disorders		112 (14.6%)	78 (15.3%)	34 (13.3%)	0.5
	ENT conditions (i.e., recurrent epistaxis, allergic rhinitis, chronic sinusitis)		25 (3.3%)	18 (3.5%)	7 (2.7%)	0.7
	Other medical comorbidities		259 (33.8%)	151 (29.5%)	108 (42.2%)	<0.001
Psychiatric	Any psychiatric comorbidity		538 (70.1%)	330 (64.6%)	208 (81.3%)	<0.001
	Depression		389 (50.7%)	239 (46.8%)	150 (58.6%)	0.002
	Suicidal attempt		52 (6.8%)	34 (6.7%)	18 (7%)	0.9
	Suicidal ideations		112 (14.6%)	68 (13.3%)	44 (17.2%)	0.1
	Anxiety		288 (37.5%)	177 (34.6%)	111 (43.4%)	0.02
	Altered mental status		36 (4.7%)	23 (4.5%)	13 (5.1%)	0.7
	ADHD		42 (5.5%)	28 (5.5%)	14 (5.5%)	0.99
	PTSD		64 (8.3%)	34 (6.7%)	30 (11.7%)	0.02
	Bipolar		61 (8%)	34 (6.7%)	27 (10.5%)	0.06
	Schizophrenia		17 (2.2%)	16 (3.1%)	1 (0.4%)	0.01
	Borderline Personality Disorder		49 (6.4%)	21 (4.1%)	28 (10.9%)	<0.001
	Antisocial Personality Disorder		9 (1.2%)	9 (1.8%)	0 (0%)	0.03
	Insomnia or hypersomnia		88 (11.5%)	54 (10.6%)	34 (13.3%)	0.3
	Eating disorder		17 (2.2%)	3 (0.6%)	14 (5.5%)	<0.001
	Conversion disorder		6 (0.8%)	3 (0.6%)	3 (1.2%)	0.4
	Disruptive behavior disorder		4 (0.5%)	4 (0.8%)	0 (0%)	0.3
	Cognitive deficit		20 (2.6%)	17 (3.3%)	3 (1.2%)	0.09
	Other Psychiatric Comorbidities		148 (19.3%)	104 (20.4%)	44 (17.2%)	0.3
Substance Use	Alcohol Use Disorder Mild		23 (3%)	16 (3.1%)	7 (2.7%)	0.4
	Alcohol Use Disorder Moderate/Severe		568 (74.1%)	388 (75.9%)	180 (70.3%)	0.1
	Cannabis Use Disorder		68 (8.9%)	51 (10%)	17 (6.6%)	0.1
	Nicotine Use Disorder		270 (35.2%)	185 (36.3%)	85 (33.2%)	0.4
	Other Substance Use Disorders		148 (19.3%)	100 (19.6%)	48 (18.8%)	0.8

ADHD, Attention Deficit Hyperactivity Disorder.

PTSD, Post Traumatic Stress Disorder.

### 3.4 Hospital course, including ICU admission, laboratory results, and mortality

Within the cohort, 16.8% (n=86) of males and 18% (n=46) of females required ICU admission (P=0.8). There was no sex difference in hospital length of stay (LOS) [median (IQR): 54.9 (68.6) hours in males vs. 52.6 (75.1) in females, P=0.7] or ICU LOS [median (IQR): 25.9 (27) vs. 21.8 (24.6) hours, P=0.23]. No sex difference was observed in BAC at the time of admission [median (IQR): 253 (215) vs. 253 (203) mg/dL, P=0.5]. There was no sex difference in serum TSH [median (IQR): 1.4 (1.4) vs. 1.4 (1.6) mIU/L, P=0.4]. Males had significantly higher ALT [median (IQR): 44 (56.2) vs. 26 (34) U/L, P<0.001], AST [median (IQR): 57.5 (60.7) vs. 39 (53) U/L, P<0.001], BUN [median (IQR): 10 (7) vs. 8 (3.8) mg/dL, P<0.001] and creatinine concentrations [median (IQR): 0.89 (0.27) vs. 0.72 (0.26) mg/dL, P<0.001]. There was no sex difference in eGFR using both the re-expressed MDRD II equation [Median (IQR) 94.2 (32.1) mL/min/1.73 m2 in males vs. 91.9 (27.5) in females, P=0.3] and the CKD-EPI equation [Median (IQR) 100.9 (25.4) in males vs. 100.8 (24.6) in females, P=0.5]. The all-cause mortality rate was 7.7% (n=59), with 8.2% (n=42) in males and 6.6% (n=17) in females (P=0.5). The median (IQR) time interval between hospitalization and death was 0.8 (1.4) years in males and 0.8 (1.5) years in females (P=0.7) (**Table 4**).

**Table 4:** Hospital course including ICU admission, laboratory results and mortality.

		All (n=767)	Males [n=511 (66.6%)]	Females [n=256 (33.4%)]	p-value
Hospital LOS (hours) [Median (IQR), Range]		54 (69.2),1.7-2030	54.9 (68.6),1.7-1638	52.6 (75.1),2.9-2030	0.7
ICU admission [no (%)]		132 (17.2%)	86 (16.8%)	46 (18%)	0.8
ICU LOS (hours) [Median (IQR), Range]		24.4 (28.8),	25.9 (27), 7-147.4	21.8 (24.6), 0.25-107.8	0.3
Blood alcohol concentration (mg/dL) [Median (IQR)]		253 (211)	253 (215)	253 (203)	0.5
TSH (mIU/L) [Median (IQR)]	All age groups	1.4 (1.4)	1.4 (1.4)	1.4 (1.6)	0.4
	Age <40 years	1.4 (1.3) (n=301)	1.4 (1.5) (n=200)	1.5 (1.3) (n=101)	0.8
	40-65 years	1.4 (1.3) (n=398)	1.4 (1.2) (n=265)	1.4 (1.5) (n=133)	0.6
	>65 years	1.6 (2.2) (n=68)	1.5 (2) (n=46)	2.2 (2.5) (n=22)	0.2
ALT Level (U/L) [Median (IQR)]		37 (54) (n=467)	44 (56.2) (n=318)	26 (34) (n=149)	<0.001
AST Level (U/L) [Median (IQR)]		54 (58) (n=431)	57.5 (60.7) (n=292)	39 (53) (n=139)	<0.001
Alkaline Phosphatase (U/L) [Median (IQR)]		85 (38) (n=459)	87 (35) (n=310)	87 (48) (n=149)	0.4
BUN (mg/dL) [Median (IQR)]		9 (7) (n=474)	10 (7) (n=322)	8 (3.8) (n=152)	<0.001
Creatinine (mg/dL) [Median (IQR)]		0.83 (0.27)	0.89 (0.27)	0.72 (0.26)	<0.001
eGFR (mL/min/1.73 m^2^) [Median (IQR)] [MDRD II equation]		92.8 (32.2)	94.2 (32.1)	91.9 (27.5)	0.3
eGFR (mL/min/1.73 m^2^) [Median (IQR)] [CKD-EPI equation]		100.8 (24.9)	100.9 (25.4)	100.8 (24.6)	0.5
Mortality	All	59 (7.7%)	42 (8.2%)	17 (6.6%)	0.5
	In hospital	2 (0.3%)	2 (0.4%)	0 (0%)	0.5
	After discharge	57 (7.4%)	40 (7.8%)	17 (6.6%)	0.7
Interval between hospital admission and death (years) [Median (IQR)]		0.8 (1.4)	0.8 (1.4)	0.8 (1.5)	0.7

LOS, Length of stay.

ICU, Intensive care unit.

ALT, Alanine transaminase.

AST, Aspartate transaminase.

TSH, Thyroid Stimulating Hormone.

### 3.5 Relationship between BAC, TSH, and eGFR

**Figure 1:**
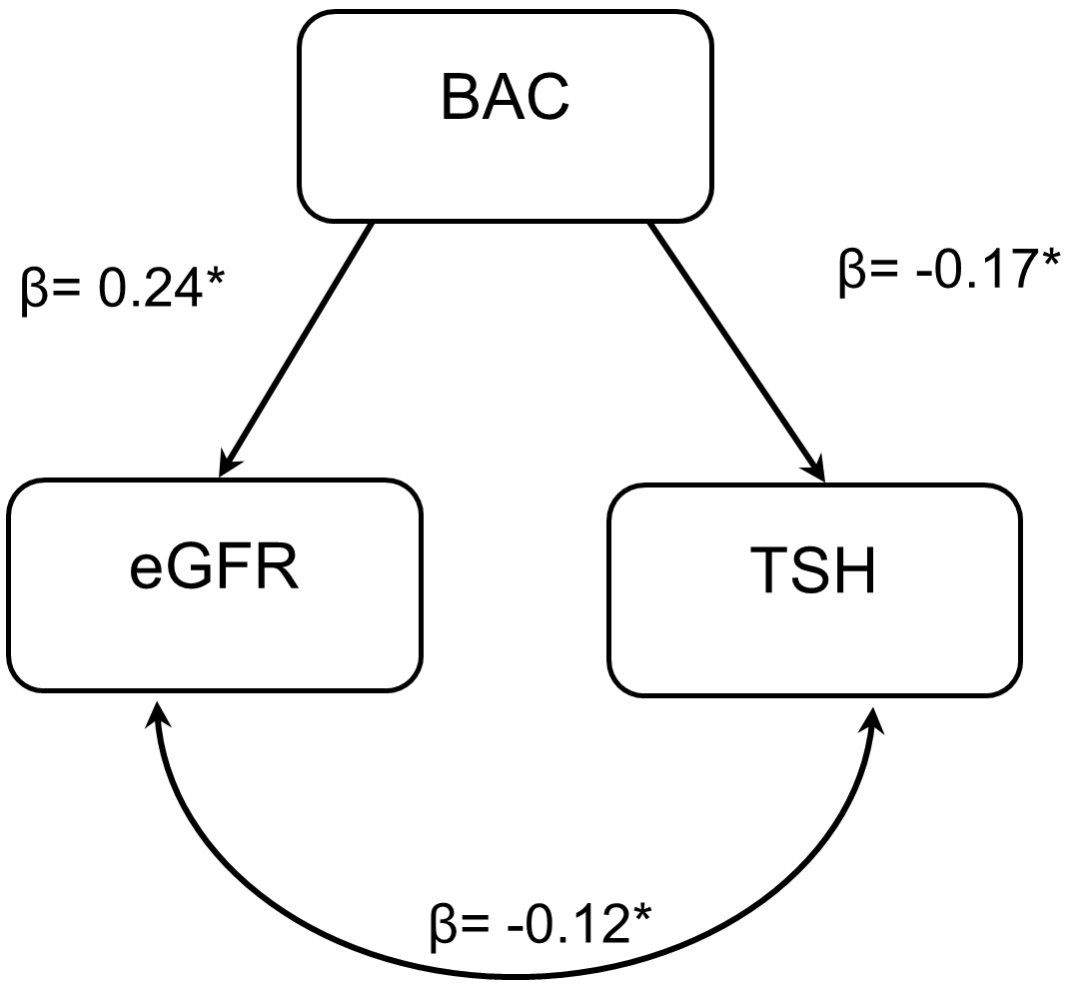
Path analysis of the BAC predicting values of eGFR and TSH via structural equation modelling. Path analyses identified a significant relationship of BAC with eGFR (CKDEPI) and TSH in males only. BAC had a positive association with eGFR (β = 0.24, p = 0.0001) and negative association with TSH (β = -0.17, p = 0.006). The covariance between the two outcomes (eGFR and TSH) was negative (β = -0.12, p = 0.049). BAC, Blood alcohol concentration; eGFR, estimated glomerular filtration rate; TSH, Thyroid-stimulating hormone. * symbol means significance.

To investigate the intricate relationship between BAC, TSH, and eGFR, we started by running a Pearson’s correlation, which showed a significant correlation between each pair in both males and females using the eGFR values calculated by the CKD-EPI equation and only in men using the eGFR values calculated by the re-expressed MDRD II equation. BAC was positively correlated with eGFR, while negatively correlated with TSH. In addition, TSH was negatively correlated with eGFR. Females showed insignificant correlation between TSH and eGFR calculated by the re-expressed MDRD II equation (**Supplementary Table S1**). Next, we ran a simple mediation analysis to test if TSH mediates the effect of BAC on eGFR, which showed TSH as a partial mediator between BAC and eGFR in males (using eGFR values of both equations) and in females (only when using eGFR values from the CKD-EPI equation) (**Supplementary Table S2**). Then, we adjusted for age, body-mass index, cardiovascular disorders, hepatic disorders, diabetes, renal disorders, and elevated liver enzymes. The partial mediation of TSH for the effect of BAC on eGFR was lost in both males and females, using eGFR values of both equations. However, the B path in males almost reached significance (P=0.052) using eGFR values from the CKD-EPI equation (**Supplementary Table S3**). To test the possible bidirectional relationship between eGFR and TSH, we further performed path analysis via SEM. Path analyses identified significant relationships between BAC with eGFR (using CKD-EPI equation) and BAC with TSH in males only (**Figure 1**). BAC had a positive association with eGFR (β = 0.24, p = 0.0001) and negative with TSH (β = -0.17, p = 0.006). The covariance between the two outcomes (eGFR and TSH) was negative (β = -0.12, p = 0.049). The eGFR re-expressed MDRD II equation had no significant path analysis in males, whereas females had no significant path analyses with either eGFR equations (**Table 5**).

**Table 5:** Path analyses of the blood alcohol concentration (BAC) predicting values of eGFR and TSH via structural equation modelling.

eGFR (CKD-EPI)					
Males			Females		
	β	p		β	p
BAC → eGFR	**0.24**	0.0001	BAC → eGFR	0.09	0.20
BAC → TSH	**-0.17**	0.006	BAC → TSH	-0.06	0.55
eGFR ↔ TSH	**-0.12**	0.049	eGFR ↔ TSH	-0.16	0.08
eGFR (re-expressed MDRD II)					
Males			Females		
	β	p		β	p
BAC → eGFR	0.22	0.0001	BAC → eGFR	0.13	0.14
BAC → TSH	-0.17	0.006	BAC → TSH	-0.06	0.55
eGFR ↔ TSH	- 0.092	0.13	eGFR ↔ TSH	-0.13	0.14

Bolded standardized coefficients denote statistical significance (p < 0.05).

## 4 Discussion

The results of this study add new insight into the rich literature documenting a significant association between alcohol consumption, thyroid state, and kidney function. Despite numerous studies on the effects of chronic alcohol use on renal function (2–5, 74–80), there remains a significant gap in the literature regarding the impact of acute alcohol intoxication on renal function. Here, we showed that alcohol has a positive effect on eGFR in males and females. Our results show that BAC can influence both eGFR and TSH, whereas eGFR and TSH can influence each other in a negative bidirectional relationship, despite which BAC still holds a positive effect on eGFR only in males. Our result of a positive effect of BAC on eGFR is consistent with several (2–5, 74–77), but not all studies (74, 78–80). A recent systematic review of 11 cohort studies (n=14,634,940) examined a potential dose-dependent association between alcohol consumption and the incidence of low eGFR of <60 mL/min/1.73 m^2^. The results showed that the incidence of low eGFR was lower in drinkers with alcohol consumption of ≤12.0 and 12.1-36.0 g/day than in non-drinkers (2). A meta-analysis of 15 cohort studies (n=268,723) reported a dose-dependent relationship between alcohol drinking and the risk of kidney damage. Specifically, patients with low (<13 g/day), moderate (13 to 26 g/day), and high (26 to 60 g/day) doses of alcohol had a 12% (RR: 0.88, 95% CI: 0.83 to 0.93), 24% (RR: 0.76, 95% CI: 0.70 to 0.83), and 21% (RR: 0.79, 95% CI: 0.71 to 0.88) lower risk of chronic kidney disease compared with the reference group (non- or occasional drinkers), respectively (5). A prospective cohort study of males followed for 14 years (n=11,023) reported an inverse relationship between moderate alcohol consumption and the risk of renal dysfunction (75). Similar results were reported in a study from Korea (n=5,729) where patients with higher alcohol intake (compared to non-drinkers) demonstrated less relative loss of eGFR over 12 years (3). In addition, the Gubbio population-based study (n=4,524) reported that alcohol intake was related cross-sectionally to eGFR and longitudinally to less negative eGFR change over time (4). Similarly, the Italian Longitudinal Study on Aging (n=3,404, aged 65-84 years) and the Southern Taiwan Pai-Wan aboriginal community study (n=1,466, aged 40-95 years) both showed an inverse linear relationship between moderate alcohol consumption and the risk of mild renal impairment (76, 77). Other studies found no effect of moderate alcohol consumption on kidney function decline (n=4,343, aged ≥65 years) (78) In addition, the Korean cohort study for outcome in patients with chronic kidney disease (KNOW-CKD, n=1,883) showed an opposite effect, where heavy alcohol consumption was associated with faster progression of CKD (79). The inconsistent results could be attributed to several confounding factors, such as comorbid liver cirrhosis. About 26% (n=219) of the patients in our cohort had comorbid hepatic conditions. We observe that BAC can influence both eGFR and TSH, and still has a positive effect on eGFR even after considering the covariates (age, body mass index, ALT and AST, diabetes, and cardiovascular, renal-urinary, and hepatic diseases) and the bidirectional relationship between TSH and eGFR. One study reported that TSH concentrations were significantly higher in patients with alcoholic liver cirrhosis (n=40) compared to healthy controls [median, range TSH=2.1 (0.4-5.3) vs. 1.1 (0.3-3.8) µU/mL, P<0.05] (18). The impact of medical and psychiatric comorbidities on the association between BAC and eGFR remains to be investigated.

We found a robust negative association between BAC and TSH in males. Alcohol has a direct toxic effect on the thyroid gland (17–19). Hermann et al. reviewed clinical studies that examined thyroid hormonal changes during chronic alcohol intake and short- or long-term abstinence and showed an overall reduction in T4 and T3 concentrations (10). TSH levels decreased in the course of chronic ethanol intake and abstinence compared to controls in some (25, 34), but not all (20, 81) studies. A few studies assessed changes in thyroid hormones during acute alcohol intoxication. Ylikahri et al. reported that single-dose alcohol administration to healthy male volunteers (n=12) had no significant effect on basal concentrations of TSH, T4, or T3 at 4 and 14 hours post-alcohol intake (82). Taken together, our study documents, for the first time, in a large sample (n=767) that acute alcohol intoxication is associated with a significant reduction in TSH in males. We further show that suppressed TSH is associated with higher eGFR.

Both hypothyroidism and hyperthyroidism have direct effects on GFR (40–42, 83). Several studies have shown that patients who receive treatment for thyroid disorders have a lower risk of developing renal dysfunction compared to patients with untreated thyroid disorders (46, 65, 84). Woodward et al. reported significantly lower median eGFRs in hypothyroid (n=550), compared to euthyroid (n=793), and hyperthyroid (n=680) patients (64 vs. 77 vs. 107 mL/min/1.73 m^2^, respectively) (85). The study by Shin et al. highlighted a significant reduction in the rate of decline in renal function among chronic kidney disease (CKD) patients with subclinical hypothyroidism (SCH) following thyroid hormone replacement therapy (THRT), with rates decreasing from -4.31 ± 0.51 to -1.08 ± 0.36 (mL/min)/(year·1.73 m2), compared to pre-THRT levels (65). In addition, patients with hypothyroidism (n=114,872) had a significantly higher risk of chronic kidney disease (CKD) compared with euthyroid individuals [OR (95%CI): 1.25 (1.21-1.29)] (86). Similar results were reported in older adults (n=1,571), where the presence of either hypothyroidism or hyperthyroidism had an 84% higher likelihood of having CKD [OR (95% CI): 1.84 (1.03-3.31)] and patients in the highest versus lowest quartile (reference) of TSH had significantly greater odds of prevalent CKD [OR (95% CI): 1.82 (1.22-2.71)] and [OR (95% CI): 1.64 (1.10-2.45)], respectively (87). Furthermore, in patients with moderate-to-severe CKD (n=461,607), an increase in serum TSH by 0.11 mIU/L was associated with a reduction of eGFR by 10 mL/min/1.73 m^2^ (95% CI: 0.10-0.11 mIU/L, P<0.001) (62) and higher TSH showed a significant negative correlation with eGFR in euthyroid individuals (n=10,859, β=-0.072, P=1.994x10^-22^) (88), with a positive association between high-normal levels of TSH and increased risk of incident CKD (n=104,633, OR (95% CI): 1.26 (1.02-1.55)] (89). In individuals with no history of thyroid disease (n=29,480), TSH within the reference range (0.50-3.5 mU/l) was negatively associated with eGFR (P<0.001). Compared with people with TSH in the lower third of the reference range ((0.50-1.4 mIU/L) eGFR was lower (83.0 mL/min per 1.73 m^2^), in people with TSH in the middle (81.6 mL/min per 1.73 m^2^) and highest third (80.3 mL/min per 1.73 m^2^) of the reference range, and in people with subclinical (79.3 mL/min per 1.73 m^2^), P<0.001) or overt hypothyroidism (76.5 mL/min per 1.73 m^2^), P<0.001) (90). Moreover, the administration of recombinant human TSH to patients after total thyroidectomy due to differentiated thyroid cancer caused a significant reduction in eGFR (64). Interestingly, the effect of thyroid status on GFR may be specific to TSH and not free T4 (FT4), as in both Zhang et al. and Wang et al. studies, there were no associations observed between FT4 and eGFR (88, 89).

The impact of thyroid dysregulation on kidney function could be mediated in part by cardiovascular and systemic hemodynamic effects (91) and by the direct effect of TSH on renal metabolism (92). Indeed, TSH receptor mRNA and protein expression are identified in the kidney (93). TSH causes an initial increase in intracellular cyclic adenosine monophosphate (cAMP) (92), followed by attenuation of the cAMP response with prolonged TSH stimulation (94). However, the effect of the thyroid on the kidney is not limited to cAMP modulation. Thyroid hormones regulate the adrenergic receptors and dopaminergic activation of the renal tubular cells (95), the water channels aquaporin 1 and 2 (96), the activity of the Na/K ATPase enzyme (97, 98), and the tubular permeability to sodium and potassium (99, 100).

Interestingly, the positive impact of BAC on eGFR with a bidirectional negative relationship between eGFR and TSH was observed only in men. Pituitary TSH receptors show a sex difference with greater density in female rats under the stimulatory effect of estrogen, while androgen has an inhibitory effect on TSH receptors (101). There is strong evidence that alcohol affects men and women differently (102), as do thyroid disorders (103). However, further studies are needed to investigate the impact of sex hormones on the complex relationship between alcohol consumption, thyroid status, and kidney performance.

The results of this study should be viewed considering both its strengths, specifically the large sample size and deep phenotyping, and its limitations. The retrospective design of our study restricts the availability of data about the pattern or duration of alcohol drinking, which could be a confounding factor. We did not consider the timing of TSH measurements, which could have impacted the results given the normal variation in thyroid hormone concentrations throughout the day (31, 32). In addition, our cohort may not be representative of the public during acute intoxication due to the high prevalence of medical and psychiatric comorbidities. The literature indicates a lower representation of females in AWS studies (104). Despite our study having a high number of female participants compared to others, the relatively lower number of female patients included may influence the generalizability of our findings. Despite these limitations, our study is the first, to the best of our knowledge, that describes sex difference in the link between acute alcohol intoxication and kidney performance despite the bidirectional negative relationship between TSH and eGFR. While our study provides valuable insight into the relationship between acute alcohol use and kidney functions, it is noteworthy that our discussions primarily focus on chronic alcohol consumption studies in the literature due to the absence of studies on acute alcohol intoxication and kidney function. Therefore, future studies evaluating acute alcohol intoxication, thyroid functions, and kidney functions are needed. The positive impact of BAC on TSH with a bidirectional negative relationship between TSH and eGFR remained consistent only with the CKD-EPI equation after adjusting for confounders. The disparity in results between these two equations warrants further investigation.

## Data availability statement

The data analyzed in this study is subject to the following licenses/restrictions: Mayo Clinic Regulations. Requests to access these datasets should be directed to abulseoud.osama@mayo.edu.

## Ethics statement

This study was approved by the Institutional Review Board (IRB) of the Mayo Clinic (ID#22-008591). The studies were conducted in accordance with the local legislation and institutional requirements. The participants provided their written informed consent to participate in this study.

## Author contributions

HU: Data curation, Writing – original draft. AY: Conceptualization, Formal analysis, Methodology, Writing – review & editing. KM: Data curation, Writing – review & editing. NM: Data curation, Writing – review & editing. AT-A: Formal analysis, Methodology, Writing – review & editing. TC: Formal analysis, Methodology, Writing – review & editing. MF: Writing – review & editing. LT: Writing – review & editing. OA: Conceptualization, Data curation, Formal analysis, Investigation, Methodology, Project administration, Supervision, Validation, Writing – original draft.
