# Supplementary material for: Bidirectional negative relationship between thyrotropin and kidney function during alcohol intoxication in males
Source: Front Nephrol. 2024 Aug 8;4:1322791. doi: 10.3389/fneph.2024.1322791 (PMC11339534; doi:10.3389/fneph.2024.1322791)
Supplement: Supplementary file 1 [file Table_1.docx]

**Supplementary Table 1S: Pearson Correlation**

**Supplementary Table 1Sa: Pearson Correlation between BAC, log TSH, and eGFR (calculated by the CKD-EPI equation) in males.**

|  |  | eGFR | Log TSH | BAC |
| --- | --- | --- | --- | --- |
| eGFR | r |  | -0.2 | 0.3 |
|  | P |  | <0.001 | <0.001 |
| Log TSH | r | -0.2 |  | -0.22 |
|  | P | <0.001 |  | <0.001 |
| BAC | r | 0.3 | -0.22 |  |
|  | P | <0.001 | <0.001 |  |

**Supplementary Table 1Sb: Pearson Correlation between BAC, log TSH, and eGFR (calculated by the CKD-EPI equation) in females.**

|  |  | eGFR | Log TSH | BAC |
| --- | --- | --- | --- | --- |
| eGFR | r |  | -0.14 | 0.17 |
|  | P |  | 0.026 | 0.006 |
| Log TSH | r | -0.14 |  | -0.14 |
|  | P | 0.026 |  | 0.024 |
| BAC | r | 0.17 | -0.14 |  |
|  | P | 0.006 | 0.024 |  |

**Supplementary Table 1Sc: Pearson Correlation between BAC, log TSH, and eGFR (calculated by the re-expressed MDRD** II **equation) in males.**

|  |  | eGFR | Log TSH | BAC |
| --- | --- | --- | --- | --- |
| eGFR | r |  | -0.2 | 0.3 |
|  | P |  | <0.001 | <0.001 |
| Log TSH | r | -0.2 |  | -0.2 |
|  | P | <0.001 |  | <0.001 |
| BAC | r | 0.3 | -0.2 |  |
|  | P | <0.001 | <0.001 |  |

**Supplementary Table 1Sd: Pearson Correlation between BAC, log TSH, and eGFR (calculated by the re-expressed MDRD** II **equation) in females.**

|  |  | eGFR | Log TSH | BAC |
| --- | --- | --- | --- | --- |
| eGFR | r |  | -0.12 | 0.16 |
|  | P |  | 0.053 | 0.01 |
| Log TSH | r | -0.12 |  | -0.14 |
|  | P | 0.053 |  | 0.024 |
| BAC | r | 0.16 | -0.14 |  |
|  | P | 0.01 | 0.024 |  |

**Supplementary Table 2S: Mediation analysis**

**Supplementary Table 2Sa: TSH as a mediator between BAC and eGFR in males (using eGFR values from the re-expressed MDRD** II **equation).**

|  | Path | Estimate | Standardized estimate (beta) | Standard error (SE) | P |
| --- | --- | --- | --- | --- | --- |
| A path | BAC → TSH | -0.000546 | -0.216105 | 0.000109 | <.0001 |
| B path | TSH → eGFR | -15.254 | -0.18385 | 3.625516 | <.0001 |
| C Path | BAC → eGFR | 0.053801 | 0.255867 | 0.009036 | <.0001 |
| C′ Path | BAC→ TSH → eGFR | 0.047649 | 0.22661 | 0.009181 | <.0001 |

**Supplementary Table 2Sb: TSH as a mediator between BAC and eGFR in males (using eGFR values from the CKD-EPI equation)**

|  | Path | Estimate | Standardized estimate (beta) | Standard error (SE) | P |
| --- | --- | --- | --- | --- | --- |
| A path | BAC → TSH | -0.000546 | -0.216105 | 0.000109 | <.0001 |
| B path | TSH → eGFR | -13.44892 | -0.19319 | 3.03042 | <.0001 |
| C Path | BAC → eGFR | 0.047285 | 0.268272 | 0.007534 | <.0001 |
| C′ Path | BAC→ TSH → eGFR | 0.041882 | 0.237615 | 0.007643 | <.0001 |

**Supplementary Table 2Sc: TSH as a mediator between BAC and eGFR in females (using eGFR values from the re-expressed MDRD** II **equation)**

|  | Path | Estimate | Standardized estimate (beta) | Standard error (SE) | P |
| --- | --- | --- | --- | --- | --- |
| A path | BAC → TSH | -0.000401 | -0.1416 | 0.000176 | 0.0237 |
| B path | TSH → eGFR | -7.751571 | -0.121514 | 3.988713 | 0.053 |
| C Path | BAC → eGFR | 0.029006 | 0.161237 | 0.011184 | 0.0101 |
| C′ Path | BAC→ TSH → eGFR | 0.026389 | 0.146688 | 0.011269 | 0.0200 |

**Supplementary Table 2Sd: TSH as a mediator between BAC and eGFR in females (using eGFR values from the CKD-EPI equation)**

|  | Path | Estimate | Standardized estimate (beta) | Standard error (SE) | P |
| --- | --- | --- | --- | --- | --- |
| A path | BAC → TSH | -0.000401 | -0.1416 | 0.000176 | 0.0237 |
| B path | TSH → eGFR | -7.49259 | -0.139984 | 3.338529 | 0.0256 |
| C Path | BAC → eGFR | 0.025767 | 0.170702 | 0.009369 | 0.006 |
| C′ Path | BAC→ TSH → eGFR | 0.023188 | 0.153619 | 0.009422 | 0.015 |

**Supplementary Table 3S: Corrected mediation analysis**

**Supplementary Table 3Sa: TSH as a mediator between BAC and eGFR in males (using eGFR values from the re-expressed MDRD** II **equation) after correction for confounders.**

|  | Path | Estimate | Standardized estimate (beta) | Standard error (SE) | P |
| --- | --- | --- | --- | --- | --- |
| A path | BAC → TSH | -0.000431 | -0.166652 | 0.000158 | 0.0070 |
| B path | TSH → eGFR | -7.946176 | -0.121707 | 5.532209 | 0.1536 |
| C Path | BAC → eGFR | 0.042637 | 0.216653 | 0.011681 | 0.0003 |
| C′ Path | BAC→ TSH → eGFR | 0.021586 | 0.119052 | 0.016158 | 0.1842 |

**Supplementary Table 3Sb: TSH as a mediator between BAC and eGFR in males (using eGFR values from the CKD-EPI equation) after correction for confounders.**

|  | Path | Estimate | Standardized estimate (beta) | Standard error (SE) | P |
| --- | --- | --- | --- | --- | --- |
| A path | BAC → TSH | -0.000431 | -0.166652 | 0.000158 | 0.0070 |
| B path | TSH → eGFR | -7.053701 | -0.108196 | 3.613568 | 0.0520 |
| C Path | BAC → eGFR | 0.040129 | 0.238137 | 0.009248 | <.0001 |
| C′ Path | BAC→ TSH → eGFR | 0.037091 | 0.220106 | 0.009329 | <.0001 |

**Supplementary Table 3Sc: TSH as a mediator between BAC and eGFR in females (using eGFR values from the re-expressed MDRD** II **equation) after correction for confounders.**

|  | Path | Estimate | Standardized estimate (beta) | Standard error (SE) | P |
| --- | --- | --- | --- | --- | --- |
| A path | BAC → TSH | -0.000120 | -0.042880 | 0.000267 | 0.6547 |
| B path | TSH → eGFR | -7.946176 | -0.121707 | 5.532209 | 0.1536 |
| C Path | BAC → eGFR | 0.022805 | 0.125774 | 0.016208 | 0.1621 |
| C′ Path | BAC→ TSH → eGFR | 0.021586 | 0.119052 | 0.016158 | 0.1842 |

**Supplementary Table 3Sd: TSH as a mediator between BAC and eGFR in females (using eGFR values from the CKD-EPI equation) after correction for confounders.**

|  | Path | Estimate | Standardized estimate (beta) | Standard error (SE) | P |
| --- | --- | --- | --- | --- | --- |
| A path | BAC → TSH | -0.000120 | -0.042880 | 0.000267 | 0.6547 |
| B path | TSH → eGFR | -6.418565 | -0.123374 | 3.791964 | 0.0932 |
| C Path | BAC → eGFR | 0.013616 | 0.094241 | 0.011147 | 0.2243 |
| C′ Path | BAC→ TSH → eGFR | 0.012632 | 0.087427 | 0.011075 | 0.2564 |
